# An investigation into *Toxoplasma gondii* at the human-livestock-wildlife interface, South Africa

**DOI:** 10.4102/ojvr.v91i1.2130

**Published:** 2024-04-18

**Authors:** Refilwe P. Bokaba, Veronique Dermauw, Darshana Morar-Leather, Pierre Dorny, Louis van Schalkwyk, Luis Neves

**Affiliations:** 1 Department of Veterinary Tropical Diseases, Faculty of Veterinary Sciences, University of Pretoria, Pretoria, South Africa; 2 Department of Biomedical Sciences, Institute of Tropical Medicine, Antwerp, Belgium; 3 Department of Agriculture, Faculty of Land Reform and Rural Development, Government of South Africa, South Africa; 4 Department of Migration, Max Planck Institute of Animal Behavior, Radolfzell, Germany; 5 Centro de Biotecnologia, Universidade Eduardo Mondlane, Maputo, Mozambique

**Keywords:** *Toxoplasma gondii*, seroprevalence, South Africa, humans, domestic animals, wildlife, interface area

## Abstract

Interface areas shared by humans, domestic and wild animals may serve as high transmission contexts for *Toxoplasma gondii.* However, knowledge about the epidemiology of *T. gondii* in such areas is currently limited. The present study assessed the seroprevalence of *T. gondii* in different hosts from Mpumalanga, South Africa. Furthermore, we investigated the local knowledge and related practices about *T. gondii* by conducting a questionnaire study in the community. Blood samples were obtained and analysed for *T. gondii* antibodies using a commercial multispecies latex agglutination kit. The seroprevalence detected in humans (*n* = 160; patients showing signs of acute febrile illness), cats (*n* = 9), chickens (*n* = 336) and goats (*n* = 358) was 8.8%, 0.0%, 4.2% and 11.2%, respectively. Seroprevalence in impalas (*n* = 97), kudus (*n* = 55), wild dogs (*n* = 54), wildebeests (*n* = 43), warthogs (*n* = 97) and zebras (*n* = 68) was calculated at 5.2%, 7.3%, 100.0%, 20.9%, 13.4% and 9.1%, respectively. The questionnaire revealed that 63.0% of household owners were subsistence farmers, and 35.9% were pet owners. A high level of female participation was found (75.3%) when compared to male participation (24.7%). The results show a low circulation of *T. gondii* in the domestic cycle and suggest the presence of possible bridges between the wildlife cycle and the surrounding domestic cycle.

**Contribution:**

The study contributes to identifying transmission patterns and risk factors of *T. gondii* within human and animal populations. This topic fits within the scope of the journal presenting original research in veterinary science, with the focus on wild and domestic populations on the African continent on a topic of universal importance.

## Introduction

*Toxoplasma gondii* (*T. gondii*) is a zoonotic protozoan parasite with a global distribution, infecting almost all mammals and bird species (Halonen & Weiss 2013). The consumption of infected meat and the ingestion or inhalation of infective oocysts from the environment are the most frequent and documented sources of infection of intermediate hosts, including humans (Brouat et al. 2018; Torrey & Yolken 2013). While most infections in humans are asymptomatic or cause mild clinical signs, toxoplasmosis can result in a severe and even fatal disease in congenitally infected foetuses and in immunocompromised hosts (Hill & Dubey 2002). The transmission of *T. gondii* between species has been studied mainly in the domestic lifecycle although circulation of the parasite in wildlife has also been documented (Pomerantz et al. 2016; Seltmann et al. 2020; Serieys et al. 2019). In the wildlife cycle of the parasite, herbivores and omnivores most commonly acquire the infection through the ingestion of oocysts from the environment while carnivores get infected via the consumption of infected prey.

Interface areas (any point of observation of two or more systems that are adjacent, in spite of the size of the systems) are spaces that are shared by humans, wildlife and domestic animals, thereby allowing interactions between the different species and their pathogens, including *T. gondii* (Berrian et al. 2016). In such areas, both domestic and wildlife feline populations (definitive hosts) have the ability to disseminate oocysts into their respective environments and can thus contribute to the dissemination of the parasite in areas shared by livestock and herbivorous or omnivorous wild species.

In South Africa, there are many areas where wildlife, domestic animals and humans converge and share resources. In previous studies, the seroprevalence of *T. gondii* has been determined in different host species in the country (Hammond-Aryee 2014; Hammond-Aryee et al. 2015a; Hammond-Aryee, Van Helden & Van Helden 2015b; Lukášová et al. 2018a, 2018b; Penzhorn et al. 2002). However, to the best of our knowledge, there are very few studies conducted in interface areas reporting on the seroprevalence of *T. gondii*. Data from such studies investigating *T. gondii* transmission dynamics would contribute to the understanding of the epidemiology of *T. gondii* in these wildlife-human-domestic interface areas with both public health and veterinary health importance. The current study aimed to determine the seroprevalence of *T. gondii* in humans, domestic and wild animals as well as assessing the knowledge of the parasite and associated practices among the human population in an interface area in South Africa.

## Research methods and design

### Study area

The study was conducted in Bushbuckridge Municipality (total area of 10 250 km^2^), Mpumalanga, South Africa, an interface area inhabited by humans, livestock and wildlife (Berrian et al. 2016). The area has a population of approximately 500 000 people and is surrounded by five conservation areas (where wildlife resides without the presence of humans or domestic animals), namely the Kruger National Park, Timbavati Game Reserve, Sabie Game Reserve, Manyeleti Game Reserve, and Bushbuckridge Nature Reserve. The majority of domestic animals in the area are chickens, goats, cattle and dogs, while there is also a small cat population (Berrian et al. 2016). Human and domestic animal habitats co-exist in this area and are separated from the wildlife habitats by fences. In South Africa, the law mandates the fencing of all conservation areas. Consequently, the word fences refer to the perimeter of the conservation areas and the surrounding municipalities. The villages selected (mentioned below) in the current study in the Mnisi community were chosen because they had more data in the population census on domestic animals. The study population consists of humans, cats, chickens, goats and wildlife species present in the Bushbuckridge Municipality interface area.

### Study design

This prevalence study consisted of three parts. Part I consisted of testing bio-banked human serum samples obtained during routine surveillance activities at Hluvukani Community Health Centre situated in the Hluvukani village in the Mnisi Community. Part II was a community study performed in four villages (Athol, Gottenburg, Tlhavekisa and Utah) in the Mnisi Community, in which household data were collected via questionnaires (*n* = 384; not all the respondents were households where blood samples from the domestic animals were collected), which documented their knowledge, attitudes and practices (KAP). Blood samples were collected from cats, chickens and goats. Part III consisted of the use of banked wildlife serum samples collected in conservation areas associated with the study area.

### Study population and sampling procedure

#### Part I: Human surveillance sampling

Archived serum samples used in the study were collected by the National Institute for Communicable Diseases (NICD). The NICD has a clinical-based surveillance team stationed at the Hluvukani Community Health Centre. The samples were collected in the context of ongoing investigations for newly emerging human pathogens (e.g. *Anaplasma phagocytophilum, Brucella spp., Leptospira spp., Toxoplasma gondii*), causing acute febrile illness. Using convenience sampling, blood samples were collected from patients meeting the following inclusion criteria: (1) patients residing in the Mnisi Community, (2) having a body temperature equal to or above 37 °C or a history of fever within the 48 h prior to visiting the clinic, (3) aged 18 years or older and (4) who are proven with a non-malaria diagnosis.

#### Part II: Community survey: Chicken, goat and cat sampling and household questionnaire

Blood samples from chickens, goats and cats were collected from four villages in the area, namely Athol, Gottenburg, Tlhavekisa and Utah. Blood from goats was drawn from the jugular vein in 5 mL plain vacutainer tubes. In chickens, blood was collected from the brachial vein from the underside of a stretched wing. The cephalic vein was used to draw blood in cats. Blood samples from cats and chickens were collected in 2 mL plain vacutainer tubes. Subsequent to collection, all blood samples were stored overnight at 4 °C. The next day, samples were centrifuged at 3500 g for 10 min, and the sera collected and stored at −20 °C until used in the serological assay.

Furthermore, a questionnaire was also conducted in households (*n* = 384) in the four mentioned villages. The questionnaire focussed on obtaining information regarding the socio-environmental and husbandry conditions of each household as well as resident knowledge about *T. gondii.*

Because of a lack of availability of household owners, subsistence farmers, pet owners (dogs and cats) and the targeted livestock and pets (in the specified villages), a convenience sampling method was performed from eligible households on every street. Owners, with animals that fitted the criterion inclusion, were chosen to participate in the study and blood samples were collected from their animals.

The inclusion criteria specified that community members participating in the survey or for animal sampling should: (1) reside in the villages of interest, (2) provide formal written consent to participate in the study acknowledging that the information or samples attained would be used in a research study, (3) be aged 18 years and older and (4) sampling from household with species of interest (only for sampling).

#### Part III: Wildlife biobank samples: Impalas, kudus, wild dogs, wildebeests, warthogs and zebras

Serum samples of selected wild species, that is, impalas (*Aepyceros melampus*), greater kudus (*Tragelaphus strepsiceros*), warthogs (*Phacochoerus africanus*), blue wildebeest (*Connochaetes taurinus*), African wild dogs (*Lycaon pictus)* and Burchell’s zebras (*Equus quagga*) were obtained from the South African National Parks (SANParks) biobank. These samples were collected in areas in and bordering the Kruger National Park (KNP), South Africa. These included the following areas: Afsaal, Barnard Grave, Biyamiti weir, Crocodile Bridge, De Cuiper, Doispane firebreak, Eco Training Camp, Greater Kruger National Park, Mashisiti, Hapi pan, Ingala, Kingfisherspruit, Klopperfontein, Levuvu Highwater Bridge South, Lindondard Metsi, Lindondard, Nwamariwa, Lower Sabie, Malelane, Maleteni, Mangala, Manyeleti, Mapungubwe, Marloth, Mashikiri, Matsisisi, Mayingani pan, Metsi, Mphongolo, Nhlangaluwe pan, Nwapi pan, Nyala pan, Orpen gate, Pafuri, Paradys Windmill, Phabeni gate, Punda, Return Africa Camp, S1/S4, Sabi Sands, Sabie Park, Satara, Shireni, Skukuza, South of Hapi Pan, Tamboti, Tshokwane, Tulamela and Xiphampana. The focus was to investigate the most common species found in the areas that included all the main feeding types (herbivores, omnivores and carnivores). These samples were also subject to the availability of serum samples in the biobank.

### Study sample size

The required sample sizes for the different groups were calculated using the following equation:

$$n_{0}=\frac{z^{2}P_{exp}(1-P_{exp})}{d^{2}},$$ [Eqn 1]

where *n*_0_ was the sample size of each species of interest, *z*, the *z*-score for the desired confidence interval (CI), *d*, the desired absolute precision and *P*_*exp*_, the expected prevalence (Cochran 1977). For non-wildlife samples, the desired CI was set at 95%, *d* at 5%, while for wildlife samples, CI was set at 90%, and *d* at 10%, with *P*_*exp*_ set at 50% for both (as the expected prevalence is unknown in South Africa). Afterwards, a correction was made for the finite population size:

$$n=\frac{n_{0}N}{n_{0}+(N-1)},$$ [Eqn 2]

with *n* = the finite-population corrected sample size, *n*_0_, the sample size calculated using the base formula, and *N*, the estimated population size in the area. Altogether, serum samples (*n* = 1275) from humans (*n* = 160), chickens (*n* = 336), goats (*n* = 358), cats (*n* = 9), impalas (*n* = 97), greater kudus (*n* = 55), warthogs (*n* = 97), African wild dogs (*n* = 54), blue wildebeest (*n* = 43) and Burchell’s zebras (*n* = 66) were serologically tested. Additionally, 384 household questionnaires were conducted.

### Laboratory analysis

To detect anti-*T. gondii* antibodies, the MAST^®^ Toxoreagent™ ID rapid latex agglutination commercial kit (Mast Group Ltd., United Kingdom) was used following the manufacturer’s instructions. The latex agglutination test (LAT) detects both immunoglobulin M (IgM) and immunoglobulin G (IgG), and it therefore cannot distinguish between chronic and acute *T. gondii* infections, but it is ideal for routine screening or seroprevalence studies (especially in epidemiological studies). Titres of 1/32 and 1/64 were used as the positive cut-off for human and animal samples, respectively.

### Statistical analysis

A descriptive statistical analysis was conducted. Seroprevalence and questionnaire data are presented in percentages with 95% CI and 90% CI. In the case of low cell counts, exact CIs were calculated. The *χ*^2^-test of independence was used to investigate the association between the presence of infection in humans and domestic animals with demographic variables (e.g., age group, village). Associating the presence of infection in wildlife species with demographic variables could not be performed because of the sample numbers for some of the variables were too low to conduct the statistical analysis. The significance level was set at 0.05. All statistical procedures were run using Statistical Package for the Social Sciences (SPSS) version 21 (IBM Corporation, Chicago, Illinois, United States [US]).

### Ethical considerations

The archived human samples obtained from the NICD have Human Research Ethics Committee (Medical, R14/49) approval from the University of the Witwatersrand with an additional Health Sciences Ethics Committee approval obtained from the University of Pretoria. The participation of the animal donors was subject to approval from the Research Ethics Committee (Faculty of Veterinary, University of Pretoria, REC036-19), and written consent from the owners of the animals from the Mnisi community, Bushbuckridge Municipality, Mpumalanga, was required. The Animal Ethics Committee (AEC, V064-18) approval was obtained for the study from the University of Pretoria. Participation and information of human participants involved in the questionnaire in the Mnisi study area were also subject to ethics approval from the Research Ethics Committee and the Faculty of Humanities (HUM015/0120), University of Pretoria. Approval was also obtained from the South African National Parks (SANParks) biobank for the use of wildlife species serum samples stored at their facilities. Approval for the National Department of Agriculture, Land Reform and Rural Development (DALRRD) Section 20 (12/11/1/1/6) biosafety and security was obtained for the samples used for the domestic and wildlife samples.

## Results

### Seroprevalence

#### Part I: Human samples

The seroprevalence of *T. gondii* in the human samples collected from the Hluvukani Community Health Clinic was estimated at 8.8% (*n* = 160, 95% CI: 4.9–14.2) (Table 1). The highest positive titre detected was 1/32. Most samples originated from subjects between the ages of 18 years and 35 years. No significant association (*p* > 0.05) was detected between the presence of infection and the categorised age groups (Online Appendix 1, Table S1).

**TABLE 1:** Seroprevalence of *T. gondii* in humans, domestic and wildlife species.

Study	Species	*n*+	*n*	%	95% CI	90% CI
Part I: Clinic	Human	14	160	8.8	4.9–14.2	-
Part II: Villages	Cat	0	9	0.0	0.0–33.6	-
	Chicken	14	336	4.2	2.3–6.9	-
	Goat	40	358	11.2	8.1–14.9	-
Part III: Conservation areas	Impala	5	97	5.2	-	2.1–10.5
	Kudu	4	55	7.3	-	2.5–15.9
	Warthog	13	97	13.4	-	8.1–20.5
	Wildebeest	9	43	20.9	-	11.4–33.7
	Wild dog	54	54	100.0	-	94.6–100.0
	Zebra	6	66	9.1	-	4.0–20.7

Note: *n*, sample size; *n*^+^, positive samples from the sample size.

CI, confidence interval.

#### Part II: Community sampling

Cats, goats and chickens were sampled within the community study. A seroprevalence of 11.2% (*n* = 358, 95% CI: 8.1–14.9) was detected in goats with the highest titre of 1/128 observed.

A seroprevalence of 4.2% (*n* = 336, 95% CI: 2.3–6.9) was detected in chickens, with the highest positive titre of 1/64. A significantly higher seroprevalence was observed in the village, Athol (16.7%, *p* < 0.01) when compared to the other villages (Gottenburg at 0.0%, Tlhavekisa at 0.0% and Utha at 2.5%).

None of the sampled cats were seropositive (CI: 0.0% – 33.6%) for *T. gondii*. All nine cats sampled were female, with eight of them from Tlhavekisa and only one cat from Athol. Two of the felines were in the age range of 6–11 months, and the remaining seven cats were between 1 year and 2 years old (Online Appendix 1, Table S1).

No significant association (*p* > 0.05) could be detected between the presence of infection and village, age group or sex in all the domestic species (Online Appendix 1, Table S1).

#### Part III: Wildlife sampling

The seroprevalence detected in the selected wildlife species was 5.2% (90% CI: 2.1–10.5) in impalas (*Aepyceros melampus, n* = 97, highest positive titre 1/2048), 7.3% (90% CI: 2.5–15.9) in kudus (*Tragelaphus strepsiceros, n* = 55, highest positive titre 1/256), 13.4% (90% CI: 8.1–20.5) in warthogs (*Phacochoerus africanus, n* = 97, highest positive titre 1/1024), 100.0% (90% CI: 94.6–100) in wild dogs (*Lycaon pictus, n* = 54, highest positive titre 1/2048), 20.9% (90% CI: 11.4–33.7) in wildebeests (*Connochaetes taurinus, n* = 43, highest positive titre 1/128) and 9.1% (90% CI: 4.0–17.2) in zebras (*Equus quagga, n* = 66, highest positive titre 1/256) (Table 1).

### Questionnaire results

Most of the participants were from Gottenburg (31.5%) followed by Tlhavekisa (25.5%), Utah (22.1%) and lastly Athol (20.8%). The majority of the participants who responded in the survey were women (289/384, 75.3%), between the ages of 18 years and 40 years (199/384, 51.8%). In all, 139 of the participants had completed secondary school education (36.2%; Table 2). Only 2.1% (8/384) of the household owners knew or had heard about toxoplasmosis.

**TABLE 2:** Socio-demographic characteristics of questionnaire participants (*n* = 384).

Variables	Frequency	
	*n*	%
**Village**		
Athol	80	20.8
Gottenburg	121	31.5
Tlhavekisa	98	25.5
Utah	85	22.1
**Gender**		
Female	289	75.3
Male	95	24.7
**Age range**		
18–30 years	110	28.6
31–40 years	89	23.2
41–50 years	73	19.0
51–60 years	51	13.3
61 years and older	61	15.9
**Education**		
No education	99	25.8
Grade 4–7	44	11.5
Grade 8–11	80	20.8
Matric	139	36.2
Higher certificate	7	1.8
Diploma	12	3.1
Degree	3	0.8
Postgraduate	0	0.0

Among the participants, 63.0% (242/384) were subsistence farmers and 35.9% (138/384) were pet owners. Pets and livestock were kept both indoors (in the house for pets and in a kraal and/or stable in the yard for livestock) and outdoors (in an enclosed yard for pets and in both the yard and outside the yard for livestock for grazing) with percentages of 79.7% (110/138) and 84.7% (205/242), respectively (Online Appendix 1, Table S2). Around one third (48/138, 34.8%) of the pet owners removed their pets’ faeces from the household by cleaning the yard and enclosures approximately three times a week. The majority of pet owners and subsistence farmers disposed of their deceased companion animal (67/138, 48.6%) or livestock carcasses (141/242, 58.3%) by throwing them away in the bush and/or river. Nearly all subsistence farmers interviewed, practised livestock slaughtering (233/242, 96.3%), with 81.4% (196/242) of these carrying out the slaughtering within their yards. All community members surveyed had access to water, but 67.7% (260/384) had to fetch water from boreholes in the villages and only 33.1% (127/384) were able to grow fruits and vegetables (Online Appendix 1, Table S2). Regarding meat consumption, most household owners preferred their meat well cooked (340/384, 81.8%) opposed to rare or medium rare.

## Discussion

The data obtained in this study reveal a prevalence range of 0% – 100% *T. gondii* antibodies in humans, domestic animals and wildlife in the interface area of Bushbuckridge Municipality in Mpumalanga. Knowledge on toxoplasmosis within the community was found to be very low as only 2.1% of the questionnaire participants had heard of or had some knowledge about the disease. A KAP study was done in Namibia, which surveyed livestock farmers and animal health practitioners (Samkange et al. 2022). The researchers found that 15.9% (10/63) of the farmers confirmed that they have heard of neosporosis or toxoplasmosis, and 58.8% (30/51) of the health practitioners had an average or higher level of knowledge of *T. gondii* (Samkange et al. 2022). The results obtained from Namibia are higher and this is probably because of the study focussing only on surveying commercial farmers and health practitioners. This population had arguably a higher literacy level and their sample size was relatively lower, whereas the current study focussed on all community members excluding animal health practitioners. Another KAP study done in Ethiopia, mainly focussed on pregnant women and animal health and medical professionals (Desta 2015). The researchers found that 5.77% (*n* = 156) of pregnant women and 33.82% (*n* = 68) of the animal health professionals have heard or read or have knowledge about toxoplasmosis (Desta 2015). The knowledge of *T. gondii* in pregnant women is also at a low percentage as the current study that could have included a number of pregnant women in the survey. Another study done in Malaysia also found a low awareness of the disease, toxoplasmosis, in farmers at 5.9% (*n* = 84) (Sadiq et al. 2021). The results above show a lack of education on toxoplasmosis within communities, and therefore awareness campaigns are needed to improve public and animal health.

In humans, a seroprevalence of 8.8% (95% CI: 4.9–40.2) was detected. A similarly low prevalence was found in Gauteng province in 2011 (Kistiah et al. 2011). In that study, seroprevalence was determined using the LAT in three groups, namely human immunodeficiency virus (HIV)-positive patients (9.8% [95% CI: 7.1%–13.4]; *n* = 376), HIV-negative patients (12.8% [95% CI: 8.9–15.8]; *n* = 376) and serum samples from a residual serum bank from a previous rubella prevalence study (6.4% [95% CI: 4.5–9]; *n* = 497) (Kistiah et al. 2011). Earlier studies in South Africa detected higher seroprevalence rates in humans, suggesting a possible decrease in prevalence with time. For instance, a study conducted in 1974 in the Transvaal area in South Africa detected a seroprevalence of *T. gondii* of 37% (*n* = 806) using indirect fluorescent antibody (IFA) (Mason, Jacobs & Fripp 1974). Another study conducted in 1978 in humans also detected higher prevalences of 18% (*n* = 698), 24% (*n* = 973) and 30% (*n* = 645) in Cape Town, Port Elizabeth and Durban, respectively (Jacobs & Mason 1978).

The seroprevalence obtained in chickens and goats was low at 4.2% (95% CI: 2.3–6.9) and 11.2% (95% CI: 8.1–40.9), respectively. Antibodies against *T. gondii* were not detected in the nine cats sampled. Previous studies in domestic animals in South Africa detected a higher seroprevalence when compared to the current study. A study in the Eastern Cape, also using a LAT, obtained a seroprevalence of 33.2% (95% CI: 25.3–41.1) in chickens (*n* = 137), 55.6% (95% CI: 47.0–64.2) in goats (*n* = 128), and 31.62% (95% CI: 22.9–40.4) in cats (*n* = 109) (Tagwireyi, Etter & Neves 2019). Hammond-Aryee et al. (2015a) found a *T. gondii* seroprevalence of 37.1%, (95% CI: 29.6–44.6) in feral cats in the Western Cape province of South Africa, 37.1% (95% CI: 29.6–44.62) and 8.8% (95% CI: 4.4–13.2) of IgG and IgM, respectively, and of 6.3% for both IgG and IgM antibodies using an indirect immunofluorescence test (IFAT) (Hammond-Aryee et al. 2015a).

Similar to the prevalence obtained in humans and domestic animals, the seroprevalence in impalas and kudus were at a lower percentage of 5.2% and 7.1%, respectively. Grazing species such as blue wildebeests (20.9%) and zebras (9.1%) had a higher seroprevalence than browsing species including impalas and kudus, which is likely because of the higher exposure of grazers to oocysts in the soil. Warthogs (13.4%) also had a higher prevalence as they are omnivores that can become infected both through grazing and by consumption of infected meat and organs. In other studies, a low seroprevalence was detected by enzyme-linked immunosorbent assay (ELISA) in blue wildebeests in Namibia (10%; *n* = 20) and in kudus from South Africa (8%; *n* = 13) (Lukášová et al. 2018a; Seltmann et al. 2020). In earlier studies, a higher seroprevalence was found in wildebeests (90%; *n* = 10), zebras (90%; *n* = 10) and warthogs (100%; *n* = 2) from Kenya using the Sabin Feldman dye test (Bakal, Karstad & In ‘T Veld 1980). Zebras (*n* = 29) from Tanzania were shown to have a seroprevalence of 28% using an IHA (Riemann et al. 1975). Although a higher prevalence of *T. gondii* antibodies was detected in these studies, they are now outdated (> 10 years old), and the sample sizes were smaller.

In the current study, a 100% seroprevalence was found in African wild dogs (carnivorous), which is in agreement with previous studies that also surveyed wild dogs in South Africa and found a prevalence of 50% and 100% using microscopy and IFAT, respectively (Hofmeyr 1956; Van Heerden et al. 1995). Unfortunately, those studies are also outdated and included small sample sizes. A more recent study investigating African wild dogs in Namibia also found a high seroprevalence (71%) using a commercial ELISA (Seltmann et al. 2020). The African wild dog mainly feeds on antelope species, which were found to be infected with *T. gondii* in this study. Studies on a variety of wild felid species such as lions, cheetahs, leopards and caracals in South Africa found seroprevalences that ranged from 50% to 100% using IFAT. From those studies, it appears that *T. gondii* infection is more prevalent in carnivores than in herbivores and omnivores. However, results have to be interpreted with caution as only a few of the studies were done on wildlife and the sample sizes were often small and not always representative of the populations (Cheadle et al. 1999; Penzhorn et al. 2002; Serieys et al. 2019). A study on honey badgers (*Mellivora capensis*) and white-tailed mongooses (*Ichneumia albicauda*) found a lower seroprevalence of 25% and 14%, respectively, but the results could be affected by predator-prey relationships and limited sample sizes (Lukášová et al. 2018a).

The low seroprevalence of *T. gondii* in humans and domestic animals detected in this study could be because of the low population of domestic cats in the sampled villages. Indeed, it is known that the presence of cats is an indicator of the presence of *T. gondii* in surrounding communities (Cheadle et al. 1999; Spencer & Markel 1993). Munday (1972) found a significantly higher seroprevalence of toxoplasmosis in sheep on islands with a cat population compared to islands with no cats. In the current study, observations during the period of sampling and the information provided by environmental monitors, veterinarians and animal technicians in the villages indicated a small cat population in the area. This also explains the small cat sample size obtained in the study. Furthermore, observations by the researchers during sampling and when the questionnaire was administered indicated that the cats in the area were free roaming. These cats did not seem to have close interactions with their owners and therefore would be easily frightened and run when approached, which could also have contributed to the limited number of samples.

Besides the absence or presence of cats, differences in prevalence between studies may be attributed to several other causes, such as climate and humidity in the area, the type of farm and husbandry practices, the feeding habits of the animals, water sources, the improper disposal of carcasses which contributes to parasite dissemination and the presence of rodent populations in the area (Boughattas et al. 2016; Halonen & Weiss 2013; Tonouhewa et al. 2017). Rodent populations can act as bridges between different ecological systems in an interface area. In our study, it was found that the overall seroprevalence of *T. gondii* in rodents in the Bushbuckridge Municipality was 18.0% (Bokaba 2019, unpublished data). The seroprevalence data from rodents also showed a seroprevalence of 19.0%, 15.3% and 20.2% in three different habitats, namely domestic, peri-domestic and a wildlife conservational area, respectively, with no statistical difference within the different habitats (Bokaba 2019, unpublished data). These data point to a continuum of infected rodent populations that interact with definitive hosts in the different ecological niches investigated. Lastly, the consumption of bushmeat (meat from wildlife animal species), husbandry and local social practices are possible pathways through which humans can become infected with *T. gondii* (Almeria et al. 2018; Ferroglio et al. 2014; Jiang et al. 2015).

In addition, the test used for serological analysis of the serum samples can affect the apparent seroprevalence. In this study, we chose a commercial LAT because of its ease of use and because this technique can be applied on a variety of species (Lappin & Powell 1991). The LAT detects both IgG and IgM antibodies and therefore cannot differentiate acute from latent infection. It is therefore intended to be used as a screening technique (Kistiah et al. 2011; Tagwireyi et al. 2019; Tuda et al. 2017). In humans, the LAT has a reported sensitivity of 94% and specificity of 100% when compared to an IFAT and a sensitivity of 86% and specificity of 100% when compared to an ELISA (Mazumder et al. 1988). Although the performance of the LAT in humans is considered to be quite satisfactory (Mazumder et al. 1988), in pigs, a lower sensitivity range of 45.9% – 47.2% and a high specificity range of 91.4% – 96.9% was reported (Dubey et al. 1995; Sroka et al. 2011). In addition, the LAT has not been validated in specifically the livestock and wildlife animal species in the study, and its application on dog serum samples has not always been successful (Ohshima, Tsubota & Hiraoka 1981).

Therefore, the results of this study should be interpreted with caution, especially those for African wild dog samples. Although the LAT test has not been validated it has been used in a number of studies in humans, domestic and wild species (Chaudhary et al. 2006; Kistiah et al. 2011; Tagwireyi et al. 2019; Taylor et al. 2008; Tuda et al. 2017).

The implementation of more advanced farming systems, the awareness of hygienic practices and the proper management of meat (how slaughtered meat is handled) could be the reasons why low prevalence rates were obtained in humans and domestic animals (Tenter, Heckeroth & Weiss 2000). Based on the questionnaire, although most participants (81%) preferred to consume their meat well cooked, which is likely to reduce the infection rate, other practices such as free-grazing, the disposal of carcasses in the bush and rivers, the dry winter season that may affect the survival of oocysts in the environment and the increase in the purchase of commercial foods in communities may contribute to *T. gondii* transmission in the study area.

## Conclusion

In conclusion, we were able to provide baseline data on the seroprevalence of *T. gondii* in humans, domestic animals and multiple wildlife species in an interface area. Nevertheless, the possible impact of the geographical proximity of these different groups on the epidemiology of *T. gondii* could not be assessed. The low prevalence found in humans and most animal species could be because of a low population of domestic cats in the area, the current culinary practice of cooking meat well, the level of hygiene in households and farms and the dry weather conditions in the area, which may affect the survival of oocysts in the environment. However, this study showed that *T. gondii* is present in the wildlife cycle, which requires further investigation focussing on prevalence, strain identification, the impact of the wildlife cycle on neighbouring habitats and whether there are overlapping bridges within interface areas. This will contribute to a better understanding of the most frequent routes of transmission and determine the impact of toxoplasmosis on public health at a provincial and national level, allowing for better disease control.

There were a few limitations in the study that might have affected the outcome. An initial limitation was the dissociation between the households that participated in the questionnaire survey and those where the sampling of livestock occurred, limiting the statistics performed. Although this was the case, the questionnaires were administered and the samples were collected in the same villages and community (Mnisi Community). Given the homogeneity of the population in cultural, social and economic terms, the results of the survey could be interpreted as a good proxy. Another limitation was the low sample size of the free roaming cats. Regarding the wildlife samples, we were limited to the SANParks biobank. Despite the above limitations, we firmly believe in the interest of our results as a contribution to the understanding of the circulation of *T. gondii* in this interface area.
